# Interplay between Structure-Specific Endonucleases for Crossover Control during *Caenorhabditis elegans* Meiosis

**DOI:** 10.1371/journal.pgen.1003586

**Published:** 2013-07-18

**Authors:** Takamune T. Saito, Doris Y. Lui, Hyun-Min Kim, Katherine Meyer, Monica P. Colaiácovo

**Affiliations:** Department of Genetics, Harvard Medical School, Boston, Massachusetts, United States of America; National Cancer Institute, United States of America

## Abstract

The number and distribution of crossover events are tightly regulated at prophase of meiosis I. The resolution of Holliday junctions by structure-specific endonucleases, including MUS-81, SLX-1, XPF-1 and GEN-1, is one of the main mechanisms proposed for crossover formation. However, how these nucleases coordinately resolve Holliday junctions is still unclear. Here we identify both the functional overlap and differences between these four nucleases regarding their roles in crossover formation and control in the *Caenorhabditis elegans* germline. We show that MUS-81, XPF-1 and SLX-1, but not GEN-1, can bind to HIM-18/SLX4, a key scaffold for nucleases. Analysis of synthetic mitotic defects revealed that MUS-81 and SLX-1, but not XPF-1 and GEN-1, have overlapping roles with the Bloom syndrome helicase ortholog, HIM-6, supporting their *in vivo* roles in processing recombination intermediates. Taking advantage of the ease of genetic analysis and high-resolution imaging afforded by *C. elegans*, we examined crossover designation, frequency, distribution and chromosomal morphology in single, double, triple and quadruple mutants of the structure-specific endonucleases. This revealed that XPF-1 functions redundantly with MUS-81 and SLX-1 in executing crossover formation during meiotic double-strand break repair. Analysis of crossover distribution revealed that SLX-1 is required for crossover suppression at the center region of the autosomes. Finally, analysis of chromosome morphology in oocytes at late meiosis I stages uncovered that SLX-1 and XPF-1 promote meiotic chromosomal stability by preventing formation of chromosomal abnormalities. We propose a model in which coordinate action between structure-specific nucleases at different chromosome domains, namely MUS-81, SLX-1 and XPF-1 at the arms and SLX-1 at the center region, exerts positive and negative regulatory roles, respectively, for crossover control during *C. elegans* meiosis.

## Author Summary

Abnormalities in the number and/or structure of the chromosomes can result in cancer, birth defects, miscarriages and infertility. In particular, the exchange of genetic material (crossover formation) between maternal and paternal chromosomes during the cell division program of meiosis is essential to produce normal sperm and eggs. Homologous recombination is the pathway utilized to make crossovers, and resolution of recombination intermediates known as Holliday junctions is the final step of homologous recombination. Four structure-specific endonucleases, MUS-81, SLX-1, XPF-1 and GEN-1, have been recently proposed to act as Holliday junction resolvases. However, how these nucleases work *in vivo* was unknown. Using *Caenorhabditis elegans* as a model system, we analyzed all possible mutant combinations for these structure-specific endonucleases. We found that XPF-1 has a redundant role with both MUS-81 and SLX-1 in promoting crossover formation. Interestingly, SLX-1 is required for proper suppression of crossovers at the center region of the autosomes. Therefore, these studies shed new light on our understanding of the mechanisms regulating both the frequency as well as the distribution of crossover recombination events during meiosis.

## Introduction

Structure-specific endonucleases are required for several kinds of DNA repair processes such as nucleotide excision repair (NER), DNA interstrand crosslink repair (ICL) and double-strand break repair (DSBR). Homologous recombination is an error free repair pathway because the broken DNA ends are repaired from templates consisting of either homologous sequence at the sister chromatids or the homologous chromosomes. During meiotic recombination, at least one DNA double-strand break has to be repaired as a crossover (obligate crossover) by homologous recombination between non-sister chromatids of a homologous pair of chromosomes. Crossover formation is essential for generating genetic diversity and promoting accurate chromosome segregation. The double (or single) Holliday junction is believed to be the intermediate required to make a crossover product [1]. The opposite sense resolution of the double Holliday junction results in crossover products, while the same sense resolution results in non-crossover products [2]. Moreover, the convergent branch migration and decatenation of such intermediates, referred to as double Holliday junction dissolution, also results in non-crossover products.

Unprocessed double Holliday junctions are toxic for cycling cells. Usually, branch migration during Holliday junction dissolution depends on the Bloom syndrome helicase (BLM) and the decatenation process is catalyzed by topoisomerase III [3]. RMI1 and RMI2 are the essential cofactors of the dissolvasome, BTR (BLM-TOP3-RMI1-RMI2) complex [4], [5]. If double Holliday junctions are not processed by the BTR complex then Holliday junction resolvases play an essential role in avoiding breaks observed at anaphase. This outcome allowed for a synthetic lethal screen with *sgs1*, which encodes the BLM helicase homolog in yeast, and identified Mus81/Slx3-Mms4/Slx2 and Slx1–Slx4 [6]. Importantly, this screening strategy did not identify Rad1-Rad10, orthologs of the human XPF-ERCC1, and Yen1, the ortholog of human GEN1, because *sgs1Δyen1Δ* and *sgs1Δrad1Δ* are viable [7]. *In vitro*, MUS81-EME1, SLX1–SLX4, and GEN1 have Holliday junction resolvase activity [8]–[14]. While the 3′flap nuclease activity of Rad1 in yeast constitutes its main function during homologous recombination [15], the XPF homolog MEI-9 is required for the majority of the crossovers formed in *Drosophila* [16].

MUS81, SLX1 and XPF require EME1, SLX4 and ERCC1, respectively, for nuclease activity. SLX4 also acts as a scaffolding protein for several DNA repair proteins including MUS81 and XPF [10]–[12]. It is reported that the *D. melanogaster* and *C. elegans* orthologs of SLX4 (MUS312 and HIM-18, respectively) are required for crossover formation during meiosis [17], [18].

In *C. elegans* meiosis, it is proposed that between 5 and 12 DSBs are evenly distributed along each pair of chromosomes [19]–[21] and one of the DSB sites is designated as a future crossover site by COSA-1, MSH-5 and ZHP-3 [22], [23]. The number of crossovers is tightly regulated as only a single crossover occurs between each homologous chromosome pair. Crossover distribution is also regulated in many organisms. For example, crossover formation is suppressed at centromeres and telomeres in budding yeast [24]. It is also known that the single interhomolog crossover is frequently located at the terminal quarters of the autosomes and the terminal thirds of the X chromosome in *C. elegans* [25], [26]. Interestingly, crossover formation is suppressed at the center of the chromosomes compared to the arm regions. Recently three studies identified mutants that showed decreased levels of crossover suppression at the center of the autosomes [21], [27], [28]. The molecular mechanisms responsible for this suppression remain to be elucidated, and one of the factors required for this crossover suppression at the center region is SLX-1 [21].

Despite the importance of Holliday junction resolution, severe meiotic defects have not been reported among single mutants for most of the structure-specific endonucleases in yeast, flies, mice or worms, with notable exceptions including *mus81* and *eme1* mutants in fission yeast, and *mei-9* in flies [9], [16]. Moreover, it is still not known whether these structure-specific endonucleases exhibit a Holliday junction resolution activity *in vivo*. To investigate whether these four structure-specific endonucleases coordinately function to form crossovers during meiotic prophase, likely as Holliday junction resolvases, we took advantage of the ease of genetic analysis and the power of high-resolution imaging in the well-defined spatial-temporal distribution of germline nuclei in *C. elegans*. We made single, double, triple and quadruple mutants of the structure-specific nucleases and analyzed phenotypes indicative of errors in chromosome segregation (decreased brood size, increased embryonic lethality, larval arrest and incidence of male offspring), as well as crossover designation, frequency and distribution, and bivalent morphology. Our studies demonstrate that: 1) HIM-18 interacts with MUS-81, SLX-1 and XPF-1; 2) XPF-1 acts redundantly with MUS-81 and SLX-1 for crossover formation; and 3) SLX-1 exhibits a region-specific crossover suppression activity. We propose that the structure-specific endonucleases coordinately function for both positive and negative control of a crossover. Moreover, this is the first report implicating the redundant actions of XPF-1 with both MUS-81 and SLX-1 in crossover formation.

## Results

### SLX-1, XPF-1 and MUS-81, but not GEN-1, interact with HIM-18/SLX-4

**Figure 1:**
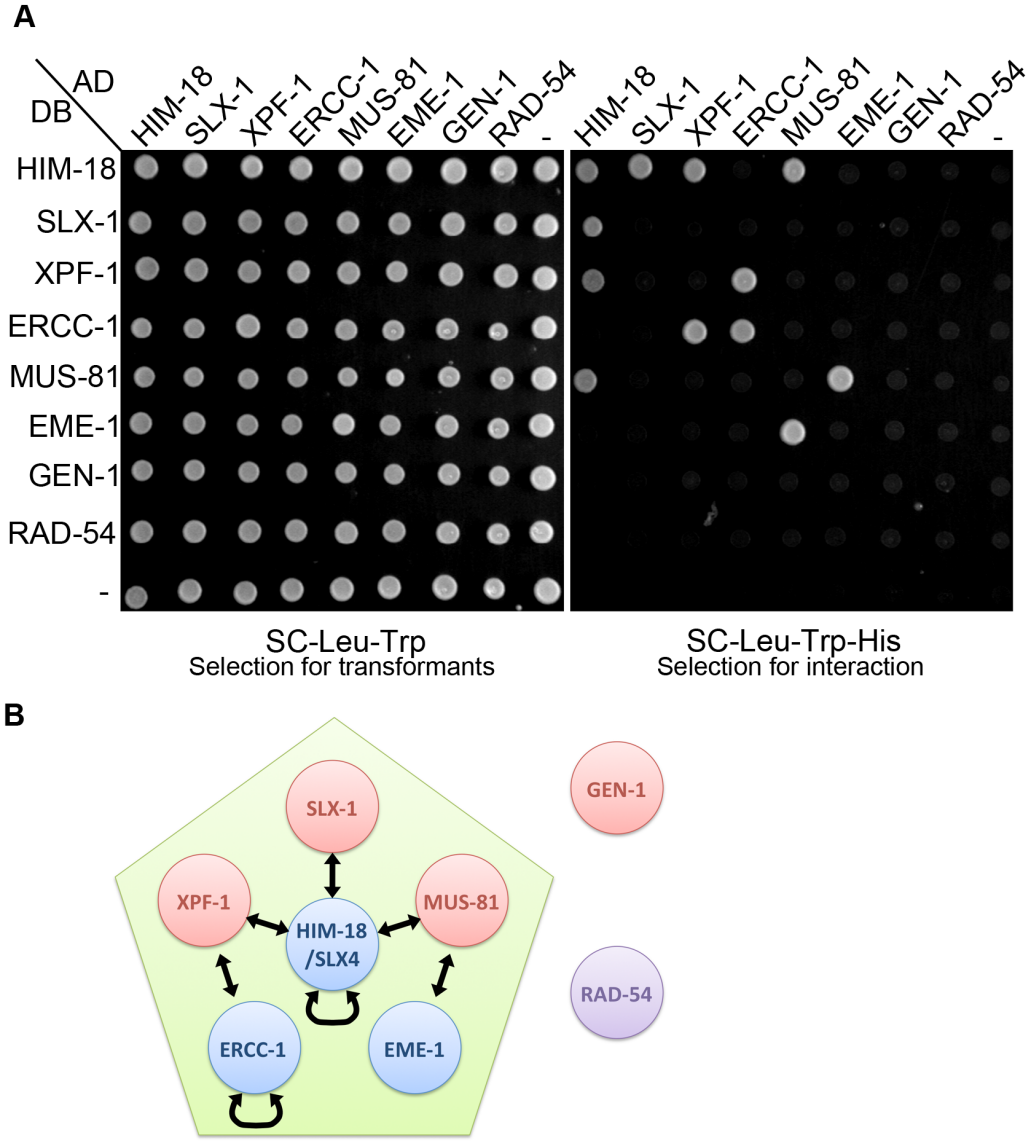
The interaction network between structure-specific endonucleases. The yeast two-hybrid system was used to examine the protein interactions between HIM-18, SLX-1, MUS-81, EME-1, XPF-1, ERCC-1, GEN-1 and RAD-54. Proteins are fused to either the DNA binding domain (DB) or the activation domain (AD) of GAL4. (A) The diploid yeast strains containing plasmids pVV212 (GAL4 DNA binding domain (DB)+*TRP1*) and pVV213 (GAL4 activation domain (AD)+*LEU2*) can grow on SC-LEU-TRP plates. All pair-wise combinations of the interactions are assayed with vector alone as the negative control. Interactions were scored on selective medium lacking leucine, tryptophan and histidine (SC-LEU-TRP-HIS). (B) Schematic representation of the interaction network. Arrows indicate protein-protein interactions.

To understand the interaction networks between structure-specific endonucleases, we performed a matrix-based yeast two-hybrid assay by using full-length constructs generated from cDNA for *him-18*, *slx-1*, *xpf-1*, *ercc-1*, *mus-81*, *eme-1* (F56A6.4), *gen-1* and *rad-54* (Figure 1). Although the ortholog of human EME1 (F56A6.4) was not previously known in *C. elegans*, we found it through a BLAST search. We detected seven protein-protein interactions, HIM-18-SLX-1, HIM-18-XPF-1, HIM-18-MUS-81, XPF-1-ERCC-1, MUS-81-EME-1, HIM-18-HIM-18 and ERCC-1-ERCC-1. We confirmed conserved interactions between each nuclease and its non-catalytic subunit, HIM-18-SLX-1, XPF-1-ERCC-1 and MUS-81-EME-1. Similar to mammals, HIM-18 interacts with three structure-specific endonucleases, namely SLX-1, XPF-1 and MUS-81. We detected two novel self-interactions: HIM-18-HIM-18 and ERCC-1-ERCC-1. These interactions support the suggestion that SLX4 and ERCC1 could make a very large (2M Dalton) protein complex in human HEK293 cells [10]. Although it is known that Mus81 interacts with Rad54 in *S. cerevisiae* [29], we did not detect this interaction in *C. elegans*. Interestingly, GEN-1 did not interact with any structure-specific endonucleases or their regulatory subunits. However, we cannot rule out the possibility that post-translational modification-dependent interactions might have been missed in a yeast two-hybrid assay. These data suggest that the structure-specific endonucleases identified thus far can be categorized into two classes, one consisting of HIM-18-associated nucleases (SLX-1, XPF-1 and MUS-81) and the second consisting of GEN-1.

### *mus-81* and *slx-1* mutants show increased sterility

**Figure 2:**
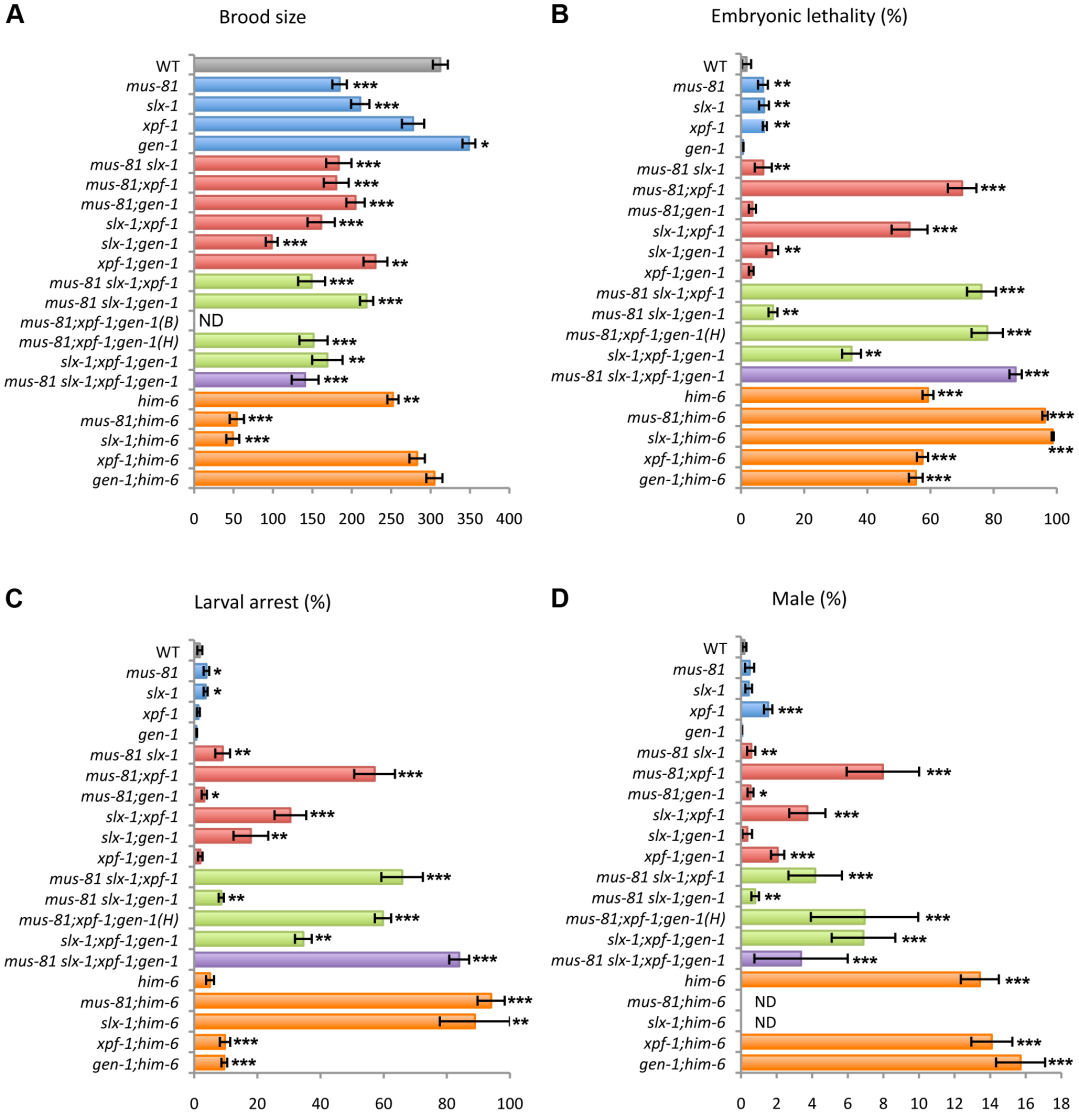
Plate phenotypes of structure-specific endonuclease mutants. (A) Brood size. Entire brood sizes were scored for singled hermaphrodites of the indicated genotypes. (B) Embryonic lethality. Total numbers of dead eggs/total number of eggs laid were scored. (C) Larval arrest. Arrested L1–L4 worms/total number of hatched worms were scored. (D) Incidence of males. Total number of adult males/total number of adult worms were scored. ND, not determined due to low N values. B, Bristol and H, Hawaiian. Error bars indicate standard error of the mean. Asterisks indicate statistical difference compared to wild type (*P<0.05, **P<0.01, ***P<0.0001, by the two-tailed Mann-Whitney Test, 95% C.I.). The total N-values and the mean values for the plate phenotypes are summarized in Table S1.

To investigate whether the structure-specific nucleases play a role in the germline, we measured the brood size in *mus-81*, *slx-1*, *xpf-1* and *gen-1* mutants (Figure 2A and Table S1). A decreased brood size is suggestive of increased sterility. The brood size was reduced to 60.5% and 68% of wild type levels in *mus-81* and *slx-1* single mutants, respectively, while there was no significant reduction in brood size for either *xpf-1* (89%) or *gen-1* (111.9%) single mutants compared to wild type. The increased sterility observed for *mus-81* and *slx-1* mutants suggests that MUS-81 and SLX-1 are required for normal germline function.

### *mus-81* and *slx-1* exhibit synthetic mitotic defects with *him-6*, the *C. elegans BLM* homolog

It is known that mutants of Holliday junction resolvases show synthetic lethality with mutants of the Bloom syndrome helicase gene in yeast and mammals [6], [30]–[32]. To investigate whether *mus-81*, *slx-1*, *xpf-1* and *gen-1* genetically interact with *him-6*, we made double mutants of each nuclease with *him-6* and examined the brood size, levels of embryonic lethality, larval arrest and the incidence of males detected among their progeny (Figure 2 and Table S1). Increases in either embryonic lethality or larval arrest are suggestive of mitotic defects. A high incidence of males (Him phenotype) is indicative of increased X chromosome nondisjunction and correlates with meiotic defects, whereas a combination of increased embryonic lethality accompanied by a high incidence of males is suggestive of increased aneuploidy resulting from meiotic missegregation of both autosomes and the X chromosome, respectively [33]. *mus-81* and *slx-1* show synthetic mitotic defects with *him-6* while *xpf-1* and *gen-1* do not. However, due to the lack of viable adult progeny, which impedes assessing the frequency of males, we cannot rule out the possibility of synthetic meiotic defects as well. These results suggest that MUS-81 and SLX-1, but not XPF-1 and GEN-1, are essential in processing recombination intermediates in the absence of HIM-6.

### *xpf-1* exhibits synergistic effects with *mus-81* and *slx-1* for embryonic and larval development as well as meiotic X chromosome disjunction

To examine whether the structure-specific nucleases play either distinct or overlapping roles in the mitotic and/or meiotic programs, we measured embryonic lethality, larval arrest and the incidence of males observed among the progeny of single, double, triple and quadruple mutants of *mus-81*, *slx-1*, *xpf-1* and *gen-1* (Figure 2 and Table S1). While only an average of 7% and 7.6% embryonic lethality was observed respectively in *mus-81* and *xpf-1* single mutants, 70% embryonic lethality was observed in *mus-81;xpf-1* double mutants (Figure 2B and Table S1). Synergistic effects were also observed regarding the phenotype of larval arrest in *mus-81; xpf-1* double mutants, where 57.1% larval arrest was observed among the surviving progeny, compared to 3.9% and 1.3% in the *mus-81* and *xpf-1* single mutants, respectively (Figure 2C and Table S1). *mus-81;xpf-1* double mutants also exhibited a higher incidence of males (8% males) among their progeny, indicative of X chromosome nondisjunction, compared to *mus-81* and *xpf-1* single mutants with 0.5% and 1.5%, respectively (*xpf-1* vs. *xpf-1;mus-81*, P<0.0025) (Figure 2D and Table S1). These results suggest that compensating activities of MUS-81 and XPF-1 are required for embryonic viability, larval development and proper X chromosome segregation.

A similar outcome is observed when examining the same phenotypes described above in *slx-1;xpf-1* double mutants compared to each single mutant (P<0.0001) (Figure 2B–2D and Table S1). These results suggest that SLX-1 and XPF-1 exhibit synergistic roles when it comes to embryonic, larval and meiotic development.

### *slx-1* and *gen-1* show synthetic sterility and larval arrest

Unlike *slx-1* single mutants, *gen-1* single mutants exhibit neither increased sterility nor increased larval arrest compared to wild type (Figure 2A and Table S1). However, the brood size of *slx-1;gen-1* double mutants decreased to 46% of *slx-1* and 28% of *gen-1* single mutants (P<0.0001) (Figure 2A and Table S1). The frequency of larval arrest observed among the progeny of *slx-1;gen-1* double mutants increased 5.3-fold compared to *slx-1* and 26-fold compared to *gen-1* single mutants (P<0.0001) (Figure 2C and Table S1). However, there is no genetic interaction between *slx-1* and *gen-1* with regard to embryonic lethality or X chromosome nondisjunction (Figure 2B–2D and Table S1). Therefore, these results suggest that SLX-1 can fully compensate for absence of GEN-1 during gametogenesis and larval development, whereas GEN-1 can only partially compensate for loss of SLX-1.

### MUS-81 and SLX-1 have redundant or compensatory roles with XPF-1 in crossover formation

**Figure 3:**
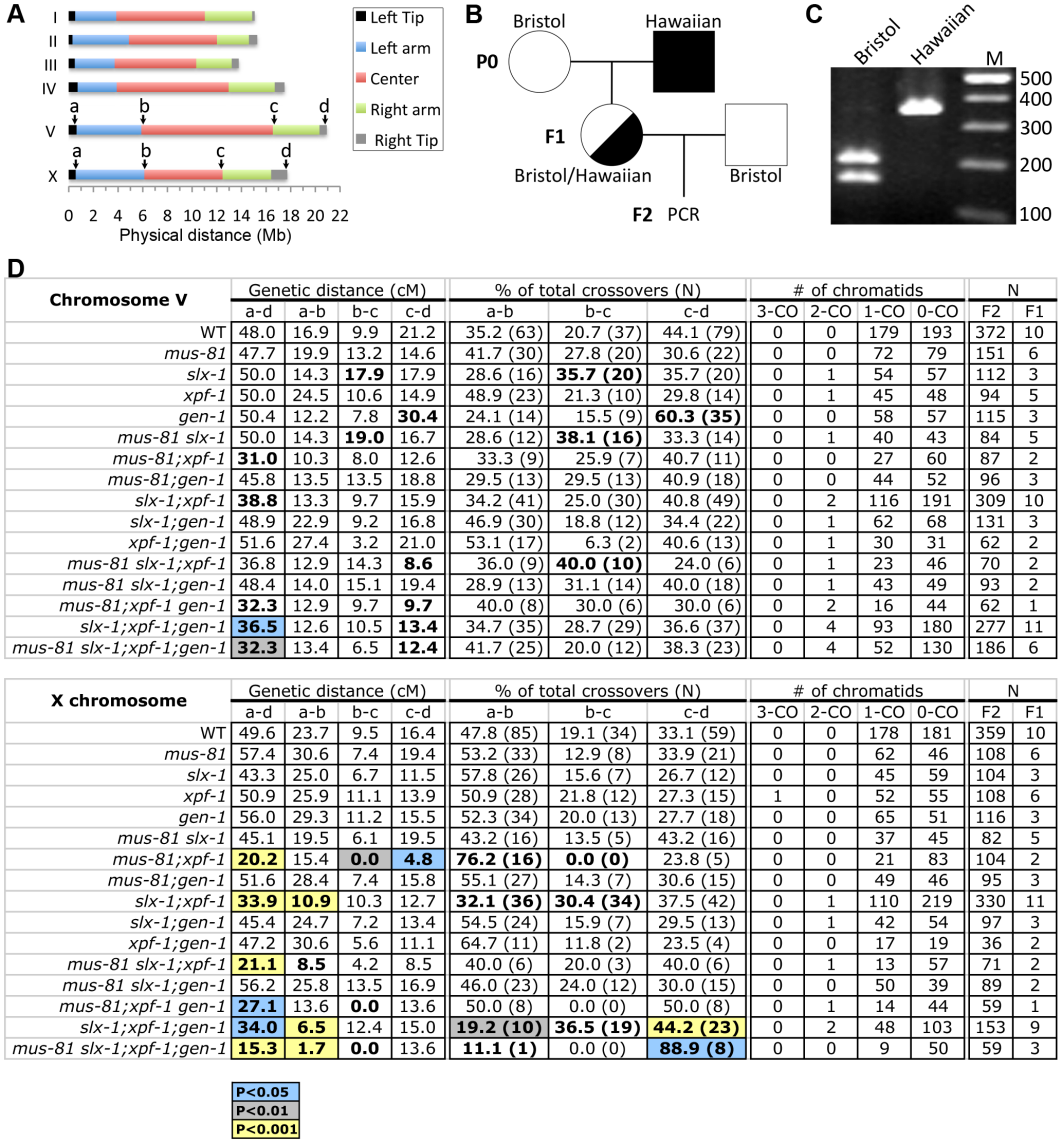
Roles for the structure-specific nucleases in regulating crossover frequency and distribution. (A) Chromosome domains. Left tip, left arm, center, right arm and right tip are indicated according to [26]. SNPs that alter the restriction length and are located closest to the boundary of each chromosome domain were chosen for chromosomes V and X. (B) Schematic representation of the strategy for obtaining the F2 progeny for snip-SNP analysis. Circles indicate hermaphrodites. Squares indicate males. White, Bristol. Black, Hawaiian. (C) The representative band pattern of PCR products at position “a” on the X chromosome. The restriction enzyme (*Bsp*HI) treatment was performed before loading. (D) Crossover frequencies and distributions observed in the entire a–d interval, left arm (a–b), center (b–c), and right arm (c–d) on chromosomes V and X. Highlighted cells indicate statistical difference compared to wild type (blue; P<0.05, grey; P<0.01, and yellow; P<0.001 by the Fisher's Exact Test and corrected by Sidak adjustment for multiple comparisons). Bold type indicates statistical significance (P<0.05) before Sidak correction.

It is known that a single crossover occurs at the terminal quarters along autosomes and at the terminal thirds along the X chromosome in *C. elegans* meiosis [25], [26]. Recently, the boundaries between different chromosome domains (tips, arms and center regions) have been reported using high-density single-nucleotide polymorphism (SNP) genotyping on a large panel of recombinant inbred advanced intercross lines (RIAILs) in *C. elegans* [26] (Figure 3A). Recombination was not observed at either the left or the right tips (∼0.5 Mb from telomeric ends) of each chromosome [26]. To examine whether the four structure-specific nucleases show either distinct or overlapping roles in meiotic crossover formation we compared crossover frequencies and distribution as in [34] along both chromosomes V and X between wild type and all single, double, triple and quadruple mutants (Figure 3). Specifically, we monitored four SNP sites located near the ends of chromosomes V and X (positions a and d), and at the boundaries between the arms and the center (positions b and c) of these two chromosomes (Figure 3A). The SNP sites selected were closely juxtaposed to the boundaries defined in the Rockman and Kruglyak study [26]. This analysis allowed us to compare the crossover frequency and distribution on the left arm, center and right arm of these chromosomes.

We assayed 48 cM and 49.6 cM intervals corresponding to 96.7% and 96.9% of the whole lengths of chromosomes V and X, respectively (Figure 3A to 3D). None of the single mutants tested exhibited a statistically significant change in the crossover frequency in this interval (interval a–d) for either chromosome compared to wild type (Figure 3D). However, we observed a significant reduction of crossover frequency in all *mus-81; xpf-1* and *slx-1; xpf-1* backgrounds (Figure 3D). Specifically, we observed the following frequencies compared to wild type on chromosomes V and X, respectively: *mus-81;xpf-1* (65% and 41% of wild type), *mus-81;xpf-1;gen-1* (67% and 55%), *mus-81slx-1;xpf-1;gen-1* (67% and 31%), *slx-1;xpf-1* (81% and 68%), and *slx-1;xpf-1;gen-1* (76% and 69%) (Figure 3D). Notably, while the following mutants exhibited a significant decrease (P<0.05) in crossover frequency when compared to wild type for the a–d interval on chromosome V: *mus-81; xpf-1* (P = 0.0041), *slx-1;xpf-1*(P = 0.0133), *mus-81 slx-1; xpf-1* (P = 0.0870, borderline significance likely due to the low N-value), and *mus-81;xpf-1;gen-1* (P = 0.0142) (Table S2), we no longer observed this significance after Sidak correction for multiple comparisons (Figure 3D and Table S3). However, this may be an artifact of the stringency of the Sidak correction as suggested by our observations of increased embryonic lethality and frequency of males in these mutants accompanied by chromosomal abnormalities in late meiotic prophase I (Figure 2 and see below). These data therefore suggest that MUS-81, XPF-1 and SLX-1 all contribute to crossover formation, with XPF-1 functioning redundantly with MUS-81 and SLX-1, respectively. This is further supported by the high embryonic lethality observed in all the *mus-81; xpf-1* and *slx-1; xpf-1*, but not *mus-81slx-1*, backgrounds (Figure 2B and Table S1). Interestingly, no further significant reduction was observed when a *gen-1* mutation was introduced into the *mus-81;xpf-1* or *slx-1; xpf-1* backgrounds. Therefore, *C. elegans* GEN-1 does not seem to be involved in crossover formation, although the accompanying study by O'Neil *et al.* [35] observed that microinjection of human GEN1 rescues the accumulation of joint molecules in *mus-81;xpf-1* double mutants.

Finally, crossover frequency on the X chromosome of *mus-81;xpf-1* double mutants is 60% of the level observed in *slx-1;xpf-1* double mutants (P = 0.0101). Furthermore, introduction of an *slx-1* mutation did not further affect the crossover frequency observed in *mus-81;xpf-1* double mutants. Thus, a *mus-81* mutation causes a more severe effect than an *slx-1* mutation in the *xpf-1* background. Taken together, these data suggest that both MUS-81 and SLX-1 have either redundant or compensatory roles with XPF-1 in regulating crossovers on both autosomes and the X chromosome.

### Regulation of crossover interference is impaired in multi-nuclease mutants

In general, only a single crossover occurs between each pair of homologous chromosomes during prophase I of *C. elegans* meiosis [36]. Therefore, crossover interference, by which the formation of an interhomolog crossover in a given chromosome region discourages formation of additional crossovers nearby, is strictly enforced in *C. elegans* [37]. Thus, the occurrence of multiple crossover events between homologous chromosomes indicates misregulation of crossover interference in this system. While we did not observe multiple crossovers in wild type for either chromosomes V or X, these were observed in multi-nuclease mutants (Figure 3D). For example, 4.1% and 7.1% of total crossover events, calculated as in [34], were double crossovers on chromosome V in *slx-1;xpf-1;gen-1* triple and *mus-81slx-1;xpf-1;gen-1* quadruple mutants, respectively. These results are consistent with the observations by Agostinho *et al.* [38] and suggest that structure-specific nucleases may be involved in the regulation of crossover interference.

### SLX-1 is required for regulation of crossover distribution

As we previously reported, crossover distribution in *slx-1* mutants shifts to the center of chromosome V (35.7% of total crossovers) where crossover formation is tightly suppressed in wild type (20.7% of total crossovers) (P = 0.0312; Fisher's Exact test) (Table S2). Notably, this statistical significance is no longer observed following Sidak correction for multiple comparisons (P = 0.3784) (Figure 3D and Table S3). However, there are additional phenotypes that can be explained, at least in part, by a deregulation in crossover distribution, such as the increased embryonic and larval lethality, decreased brood size, and increased chromosome abnormalities (see below) observed in *slx-1* single mutants. In addition, a similar shift was observed in Agostinho *et al.* [38]. These results suggest that SLX-1 exhibits either anti-crossover activity or pro-non-crossover activity at the center region of the autosomes.

The increased crossover levels previously detected on the center of the X chromosome in *slx-1* mutants compared to wild type [21] were not recapitulated here. This is due to the interval previously used in this analysis, which included regions of both the center and the right arm of this chromosome. In our current study, the more strictly defined SNP sites at the boundaries of the arms and the center, allow us to observe a more precise crossover distribution in each chromosome domain and to correlate our findings to the global analysis presented for wild type in Rockman and Kruglyak [26]. Therefore, we conclude that SLX-1 does not inhibit interhomolog crossover formation at the center region of the X chromosome.

### Crossover designation is not altered in most endonuclease-defective mutants

ZHP-3, the *C. elegans* homolog of *S. cerevisiae* Zip3, is a crossover-promoting factor [23], [39]. ZHP-3 initially localizes along the length of chromosomes, but becomes restricted to six distinct foci per nucleus in late pachytene, marking the six crossover precursor sites (one per homolog pair) [23], [39]. Since SNP analysis revealed that crossover frequency was reduced in *mus-81;xpf-1* and *slx-1;xpf-1* double mutants, we assessed the number of ZHP-3 foci per nucleus in the nuclease-defective mutants to determine if a similar reduction in ZHP-3 foci could be observed (Table 1). All single mutants, as well as all double, triple, and quadruple nuclease mutant combinations did not result in changes in the number of ZHP-3 foci (∼6 per nucleus). This is consistent with the observed number of ZHP-3 foci in the single nuclease-defective mutants and the double nuclease-defective mutants by Agostinho *et al.* and O'Neil *et al.* [35], [38]. Thus, the reduction in crossover frequency observed for the *mus-81;xpf-1* and *slx-1;xpf-1* backgrounds by SNP analysis could not be detected by cytological crossover analysis based on scoring the number of ZHP-3 foci per nucleus. This outcome suggests that crossover designation is not drastically affected in the nuclease-defective mutants, but that subsequent resolution of those events into crossovers is impaired.

**Table 1:** ZHP-3 foci in late pachytene and diplotene nuclei of the nuclease-deficient mutants.

strain	average # of ZHP-3 foci per nucleus	standard deviation	number nuclei (n)	Mann-Whitney P-value (nominal)	Mann-Whitney P-value (corrected)
WT	6.14	0.92	158	(ref.)	(ref.)
*mus-81*	6.00	0.97	103	0.16	0.91
*slx-1*	5.79	1.00	102	0.004	0.057
*xpf-1*	5.89	0.79	103	0.03	0.39
*gen-1*	6.09	0.64	100	0.57	>0.99
*mus-81 slx-1*	6.04	0.97	104	0.54	>0.99
*mus-81; xpf-1*	5.98	1.31	100	0.43	>0.99
*mus-81; gen-1*	6.03	0.89	108	0.47	>0.99
*slx-1; xpf-1*	6.33	1.07	108	0.26	0.98
*slx-1; gen-1*	6.08	0.96	115	0.63	>0.99
*xpf-1; gen-1*	6.14	1.13	118	0.76	>0.99
*mus-81 slx-1; xpf-1*	6.08	1.05	104	0.55	>0.99
*mus-81 slx-1; gen-1*	6.05	0.93	104	0.33	>0.99
*mus-81 xpf-1; gen-1*	ND				
*slx-1; xpf-1; gen-1*	6.18	1.29	91	0.96	>0.99
*mus-81 slx-1; xpf-1; gen-1*	6.25	1.04	100	0.44	>0.99

### The absence of structure-specific endonucleases results in severe chromosomal abnormalities

**Figure 4:**
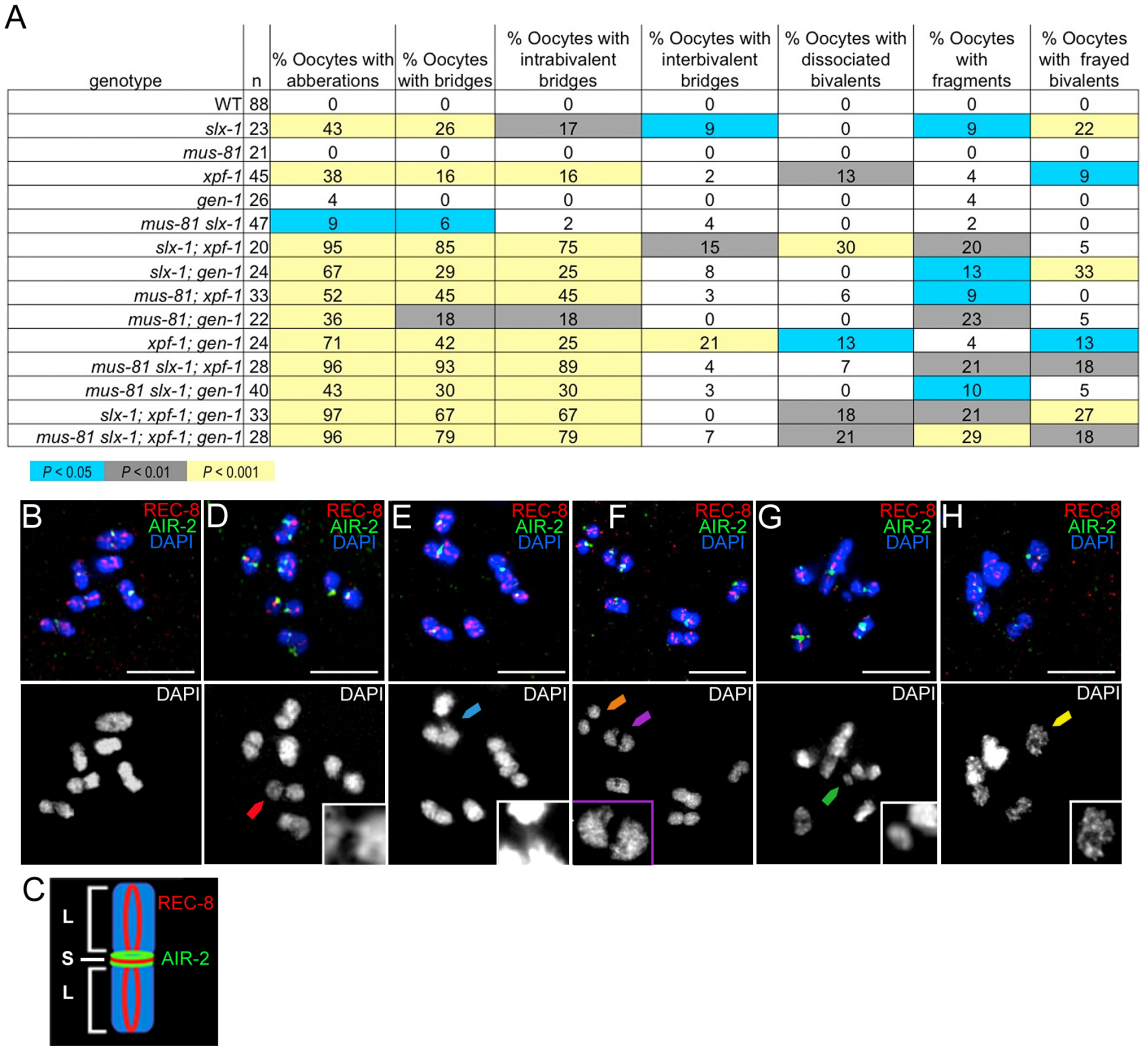
Oocytes in structure-specific endonuclease mutants exhibit chromosomal abnormalities. (A) Percentage of oocytes carrying chromosomal abnormalities in the nuclease-deficient backgrounds. Each oocyte is scored for the absence of defects or the presence of either a single or multiple chromosomal defects. Shading within the chart indicates level of statistical significance compared to wild type by the Fisher's exact test. (B) A wild type −1 oocyte at diakinesis has no observable chromosomal defects by analysis of REC-8 (red) and AIR-2 (green) immunolocalization and DAPI-stained chromatin (blue). (C) The illustration shows a single cruciform bivalent comprised of a short and long arms and depicts the normal localization of REC-8 and AIR-2. (D) A chromatin bridge between homologs (red arrowhead) is observed in a −1 oocyte from an *slx-1; xpf-1* double mutant germline. Inset shows magnified image of the intrabivalent chromatin bridge. (E) An example of an interbivalent chromatin bridge (blue arrowhead) in a −1 oocyte from an *slx-1;xpf-1* double mutant. The interbivalent chromatin bridge is shown at a higher magnification in the inset. (F) Two pairs of homologs prematurely separated at their short arms (orange and purple arrowheads) observed in a −1 oocyte from an *slx-1;xpf-1* double mutant. The separated homologs (dissociated bivalents) are identified by the uncoupling of the two AIR-2 rings (green), normally observed at the region of contact between the short arms, and the lack of observable DAPI-staining chromatin between the separated short arms. Purple arrowhead points to the dissociated bivalent magnified in the inset. (G) One DNA fragment (green arrowhead) has separated from chromosomes in a −1 oocyte from an *slx-1* mutant germline. Inset depicts a higher magnification of the DNA fragment indicated by the green arrowhead. (H) A −1 oocyte from an *slx-1;gen-1* germline with frayed bivalents. The inset shows a magnified single frayed bivalent from the oocyte. Scale bars, 5 µm. Merged images are shown with separated channels in Figure S1.

Increased sterility, embryonic lethality, and X chromosome nondisjunction in the absence of the MUS-81, XPF-1, SLX-1, and GEN-1 endonucleases indicate defects in meiotic chromosome segregation. To observe the meiotic chromosomal defects, we examined chromosome morphology in −1 oocytes at diakinesis and +1 oocytes with chromosomes at anywhere from prometaphase I to anaphase I in the endonuclease mutants (Figure 4A–H). Following formation of the single off-centered crossover between homologous chromosomes, the bivalents remodel around the crossover site adopting a cruciform-structure comprised of a short and long arms (Figure 4C) [40]. To monitor bivalent morphology, and precisely examine homolog attachment, we stained oocytes with DAPI and antibodies recognizing the meiosis-specific α-kleisin REC-8 and the aurora B kinase AIR-2 [41], [42]. REC-8 localizes along both the long and short arms during diakinesis, and is removed at the short arm at anaphase I, thereby allowing homologs to segregate to opposite poles of the spindle [41]. AIR-2 localizes as two rings only along the short arm of the bivalents until the metaphase to anaphase I transition and it has been proposed to phosphorylate REC-8 along the short arm, thus promoting its turnover [18], [43]. The bivalents in the endonuclease-defective mutants exhibited a range of chromosome defects suggestive of impaired DSBR and/or lack of mature interhomolog crossover formation, which included chromatin bridges, premature homolog separation, DNA fragments, and a frayed appearance (Figure 4) [18], [44]–[48]. The *xpf-1* and *slx-1* single mutants exhibited elevated numbers of oocytes carrying chromosomal aberrations (38% (17/45) and 43% (10/23); *P*<0.0001, respectively; Figure 4A), whereas these were not drastically increased in the *mus-81* and *gen-1* single mutants (0% (0/21), *P* = 1, and 4% (1/26), *P* = 0.23, respectively). However, all double, triple, and quadruple nuclease mutant combinations showed an increase in the frequency of oocytes with chromosomal abnormalities compared to wild type (Figure 4A). Thus, the absence of either SLX-1 or XPF-1 individually increases the frequency of oocytes with aberrations and any combination of mutations in *slx-1*, *xpf-1*, *mus-81*, or *gen-1* also results in defective bivalent morphology.

Analysis of chromosomes in the oocytes in the nuclease-defective mutants revealed chromatin bridges within bivalents (intrabivalent) and between bivalents (interbivalent; Figure 4). With the exception of the *mus-81* single mutant (0%; 0/21) and the *gen-1* single mutant (0%; 0/26), all single, double, triple, and quadruple nuclease mutants had higher than wild type levels (0%; 0/88) of chromatin bridges (intrabivalent and/or interbivalent), observed in anywhere from 6% to 93% of oocytes. Among the other single nuclease mutants (*xpf-1* (16%; 7/45) and *slx-1* (26%; 6/23)), the *slx-1* mutant had the highest frequency of oocytes with chromatin bridges within bivalents and/or between bivalents. The double mutant combinations with the highest frequency of oocytes with chromatin bridges included: the *slx-1;xpf-1* (85%; 17/20) and the *mus-81; xpf-1* mutants (45%; 15/33). Interbivalent chromatin bridges were observed in the *slx-1* single mutant (9%; 2/23), *slx-1;xpf-1* double mutant (15%; 3/20), and the *xpf-1;gen-1* double mutant (21%; 5/24). We hypothesize that the chromatin bridges between bivalents arise from multichromatid strand invasions that can be removed by nucleases or are typically prevented by helicases. High levels of oocytes with chromatin bridges have also been observed for these double mutants in the accompanying studies by Agostinho *et al.* and O'Neil *et al* [35], [38].

The *xpf-1* mutation tended to increase the frequency of oocytes where homologs have dissociated prematurely, as indicated by bivalents that have been separated at the short arm and confirmed by the presence of uncoupled AIR-2 rings (6%–30%; Figure 4). Separation at the short arm likely indicates that fragile chromatin connections between homologs have been broken. With the exception of the *mus-81; xpf-1* double mutant and *mus-81 slx-1; xpf-1* triple mutant, all strains lacking XPF-1 had a statistically significant increased level of dissociated bivalents compared to wild type (Figure 4A). The *mus-81; xpf-1* and *mus-81 slx-1; xpf-1* mutants had a borderline increase in prematurely dissociated homologs (*P* = 0.079 and *P* = 0.063, respectively).

The *slx-1* single mutant (9%; 2/23), most double mutant combinations, all triple mutants, and the quadruple mutant had DNA fragments in 9% to 29% of oocytes (Figure 4). The *mus-81 slx-1* and *xpf-1*; *gen-1* double mutants were the only mutant combinations that did not exhibit increased DNA fragments compared to wild type (*P* = 0.35 and *P* = 0.22, respectively).

Thus, absence of XPF-1, SLX-1, or combinations of any two of the four nucleases generally increased the occurrence of chromosomal abnormalities, which is consistent with the genome instability revealed by their concomitant increase in sterility, embryonic lethality, and male progeny. These plate phenotypes likely resulted from failure to properly process recombination intermediates without those endonucleases as apparent by the aberrant bivalent morphology. The nuclease-defective mutants were prone to contain oocytes with chromatin bridges, which supports the role of these nucleases in joint molecule resolution.

## Discussion

Our goal was to uncover the functional overlap and differences between the structure-specific nucleases MUS-81, SLX-1, XPF-1 and GEN-1 in promoting genomic stability in the *C. elegans* germline. We found that HIM-18/SLX-4 interacts with SLX-1, XPF-1 and MUS-81 by yeast two-hybrid. Thus, HIM-18/SLX-4 in *C. elegans* may act as a platform for the three different nucleases to regulate their activity, as previously shown *in vitro* for the human homologs [10], but it is unknown if their interactions with HIM-18/SLX-4 are mutually exclusive. We show that *xpf-1* is synergistic with either *mus-81* or *slx-1* for chromosome nondisjunction as detected by plate phenotyping (embryonic lethality, sterility, larval arrest and frequency of males), SNP mapping of crossover frequency and distribution, and the assessment of bivalent abnormalities such as chromatin bridges. This is evidence that MUS-81 and SLX-1 may act in the same pathway, which is parallel to that of XPF-1, to form crossovers and resolve chromatin bridges. Specifically, MUS-81 and SLX-1 may cleave the same recombination intermediates but their nuclease activity cannot be replaced by XPF-1 because of their potentially distinct substrate preferences. From plate phenotyping, we found that *mus-81* and *slx-1*, but not *xpf-1*, exhibit synthetic mitotic defects with *him-6*, albeit we cannot rule out the possibility of synthetic meiotic defects as well; we infer that MUS-81 and SLX-1 may cleave recombination intermediates if they are not dissolved by HIM-6. Similar to yeast Yen1, *C. elegans* GEN-1 appears to have a minor role in promoting genomic stability that is revealed only in the absence of other nucleases; the *gen-1* mutation can enhance sterility, larval arrest, and bivalent morphology defects. Here we find additional evidence supporting our previous study that SLX-1 reduces crossovers in the center of chromosomes. Although XPF-1 with MUS-81 or SLX-1 are nearly essential for progeny viability, we found that the quadruple nuclease mutant still retains some crossovers, a subset of which are no longer subject to interference. Thus, crossovers resolved by other nucleases may not be regulated by the mechanism that reinforces crossover distribution and positioning. We propose a model in which coordinate action between MUS-81, SLX-1 and XPF-1 promotes crossovers at the arms and presence of SLX-1 inhibits crossovers at the center region of chromosomes.

### In addition to MUS-81, SLX-1 and XPF-1 other candidate nucleases are involved in crossover formation

**Figure 5:**
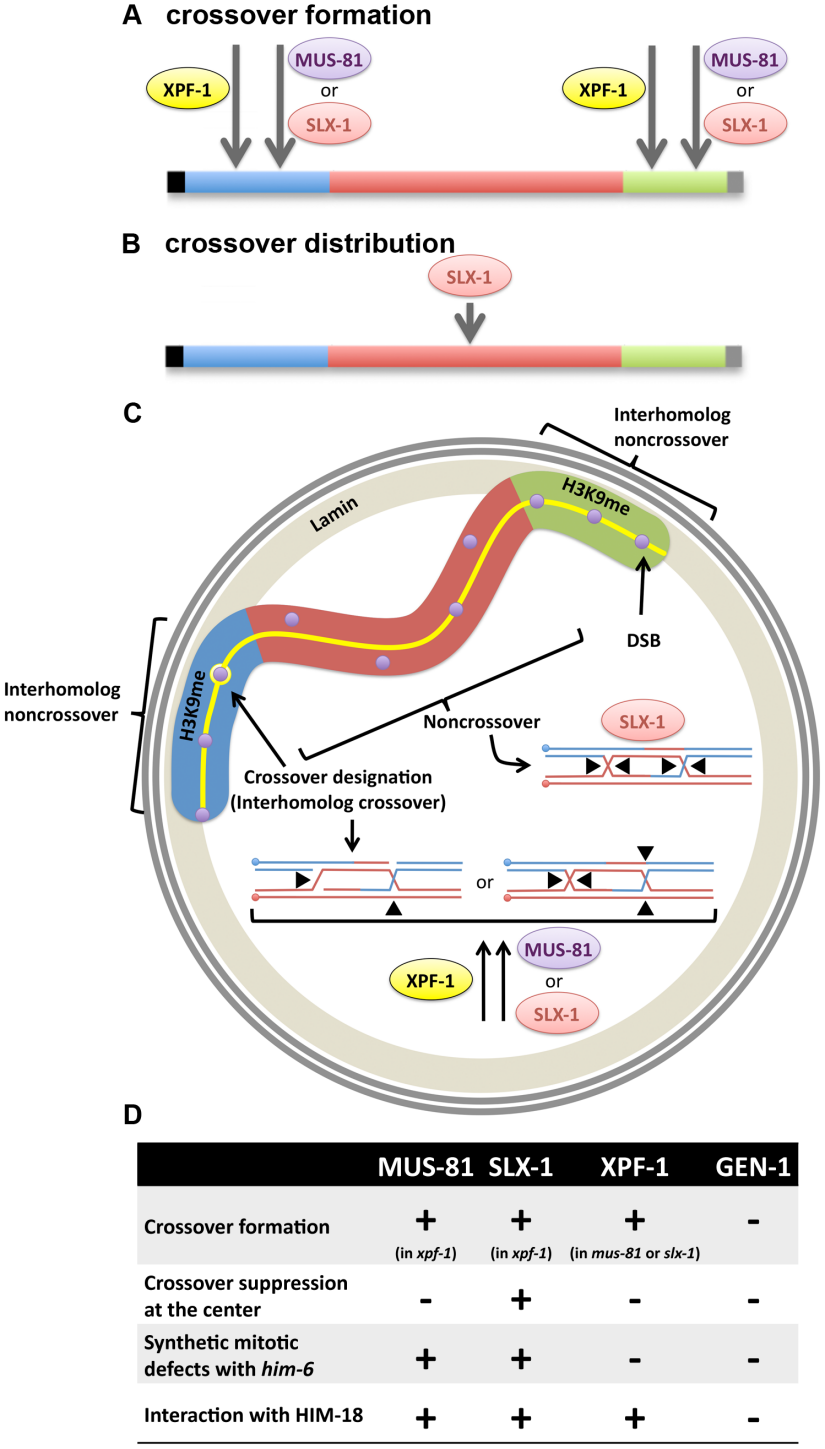
Interplay between structure-specific endonucleases for crossover control. (A) Obligate crossover regulation (assurance). MUS-81, SLX-1 and XPF-1 are factors contributing to obligate crossover formation at the arm region of the chromosome. (B) Regulation of crossover (CO) distribution. SLX-1 is important for proper crossover distribution. (C) Hypothetical spatial control of meiotic recombination. H3K9me enriched arm regions (blue and green) of the chromosome are tethered to the nuclear membrane by the transmembrane protein LEM-2. There is supposed to be an interhomolog bias at the arm regions, given that DSBs are more prone to result in interhomolog crossover formation on these regions. One of the DSBs (purple circles) at one of the arm regions is designated as the future crossover site by pro-crossover factors ZHP-3, MSH-4, MSH-5 and COSA-1 (yellow circle). At the crossover-designated site, either (1) cooperative nicking of the D-loop and the half junction or (2) opposite sense resolution of the double Holliday junction, may result in a crossover product. We propose that XPF-1 redundantly functions with MUS-81 and SLX-1, respectively, to resolve those joint molecules. The other DSBs at the arm region may be repaired by SDSA or dHJ dissolution to make noncrossovers. At the center region (red), where there is less interhomolog bias than on the arm regions, there may be a higher incidence of intersister repair (indicated by purple circles not superimposed onto the synaptonemal complex, represented by the yellow line). If double Holliday junctions are formed between homologous chromosomes, SLX-1 may promote noncrossover formation by same sense resolution of the double Holliday junction in that chromosome region. (D) Crossover phenotypes (crossover formation and distribution), synthetic mitotic defects with *him-6* and physical interaction with SLX4/HIM-18 are summarized for each structure-specific endonuclease.

Based on our crossover analysis, MUS-81, SLX-1 and XPF-1 are factors contributing to obligate crossover formation (Figure 5A). However, because *mus-81slx-1; xpf-1; gen-1* quadruple mutants still show 67% and 31% of crossovers on chromosomes V and X, respectively, this suggests that additional nucleases involved in meiotic crossover formation may exist in *C. elegans*. Recently, it has been shown that the biochemically characterized resolvases, Yen1, Mus81-Mms4, Slx1–Slx4, the Bloom syndrome helicase homolog Sgs1 and a mismatch repair complex, Exo1-Mlh1-Mlh3, are required for joint molecule resolution in yeast [49]. Although *exo-1* and *mlh-1* single mutants are viable in *C. elegans* (T. Saito et al., unpublished results and [35], [50], [51]), we cannot eliminate the possibility that EXO-1 and MLH-1 may act in a redundant manner during crossover formation in this organism. There is no MLH-3 ortholog in *C. elegans*; however, we have found that its potential nuclease motif, DQHA*X*_2_E*X*_4_E, is conserved in PMS-2 [52]. Furthermore, FAN-1, which interacts with MLH-1 and is required for interstrand crosslink repair, may also act as a Holliday junction resolvase because the VRR_NUC domain, which is conserved in the FAN-1 homolog of the archaeon *Sulfolobus solfataricus*, can cleave Holliday junctions [53].

Another aspect to consider, regarding Holliday junction resolution, is that it is reminiscent of the decatenation activity exhibited by the type I topoisomerase. This is supported by reports that the vaccinia virus topoisomerase and the human topoisomerase I can resolve synthetic Holliday junctions [54], [55]. Thus, it will be interesting to investigate whether the *C. elegans* topoisomerase family of proteins can cleave artificial Holliday junctions *in vitro* and whether they are required for crossover formation *in vivo*. In addition, the SLX4/HIM-18 complex remains a possible candidate because recently another nuclease, the human SNM1B/Apollo, was found to interact with SLX4 [56]. The SNM1B homolog in *C. elegans* is MRT-1, which is required for DNA crosslink repair and telomerase activity [57]. Finally, LEM-3/Ankle1, a protein that contains an Ankyrin repeat, LEM domain (for lamina-associated polypeptide, emerin, MAN1 domain) and a GIY-YIG type nuclease domain that is also found in SLX-1, has been recently identified. LEM domain-containing proteins connect the nuclear membrane and chromatin. Chromatin immunoprecipitation analysis (ChIP-on-chip and ChIP-seq) revealed that histone H3K9me and LEM-2 are enriched at the arm regions of the chromosomes, where crossovers are frequently observed [58]–[60]. Further studies will therefore aim to identify the additional crossover-specific Holliday junction resolvases operating during meiosis.

### Roles of SLX-1 in regulating crossover positioning

It has been reported that XND-1, HIM-5 and SLX-1 are required for the suppression of crossovers at the center of the autosomes [21], [27], [28]. However, the molecular mechanism for this chromosome region-specific crossover suppression is unknown. A possible mechanism for crossover suppression may involve same sense resolution of double Holliday junctions at the center of chromosomes by SLX-1 (Figure 5B and 5C). In addition, it is known that there are epigenetic differences between the arms and the center region of chromosomes in *C. elegans* (Figure 5C) [60]. Specifically, histone H3K9 me1/2/3 is enriched at the arm regions and H3K4me3 is enriched at the center in embryonic and larval stages. Whether this kind of epigenetic mark is maintained during meiotic recombination in the mature germline (adult worms) remains to be determined. Given the presence of a PHD-finger in SLX-1, it remains to be tested whether SLX-1 may act as a region specific crossover suppressor in part by recognizing these or other epigenetic marks defining chromosome domains or boundaries.

### A model for biased crossover resolution of Holliday junctions during meiosis

How is crossover formation regulated in *C. elegans* meiosis? It has been previously estimated that the number of DSBs is around 5–12/homologous chromosome pair during meiotic prophase in *C. elegans* [19]–[21]. Therefore, it remains unclear how only one of these DSBs is destined for repair as an interhomolog crossover while all other DSBs are repaired as noncrossovers including interhomolog noncrossovers, intersister crossovers and intersister noncrossovers.

If there is no bias in how either a single or double Holliday junction is resolved, the expectation is that the crossover/noncrossover ratio should be 1∶1. However, only opposite-sense resolution of a double Holliday junction allows for crossover formation. Since crossovers are essential during meiosis, biasing factors that reinforce opposite-sense resolution must exist to ensure crossover formation. COSA-1, MSH-5 and ZHP-3 are factors that promote crossover formation but it is not known if their biochemical activities directly promote opposite-sense resolution. Based on our observations, Holliday junction resolvases do not play a role in designating a single DSB as the site destined for repair as an interhomolog crossover (Table 1).

One possible hypothesis to explain the single crossover at one of the arm regions is that there are both interhomolog and noncrossover biases operating at the arm regions (Figure 5C). Once one of the DSBs at the arm region is marked by pro-crossover factors, such as ZHP-3, MSH-4, MSH-5 and COSA-1, the designated DSB site may undergo resolution by the redundant activities of HIM-18-binding nucleases, SLX-1, MUS-81 and XPF-1. A double nicked Holliday junction cleaved by Mus81-Eme1 in yeast [61] has been suggested to only result in a crossover. In *C. elegans*, SLX-1, XPF-1 or MUS-81 may act on a recombination intermediate consisting of a D-loop and a half junction, which resembles two nicked Holliday junctions, to form a crossover (Figure 5C). Further studies will reveal the biochemical activities of these proteins in more detail. Given the role of RTEL-1 in catalyzing D-loop disruption in vitro [48], we propose that all undesignated DSBs at the arm regions are converted into noncrossover products via an RTEL-1-dependent SDSA pathway.

In budding yeast, plants and mice, the MSH4-MSH5-dependent crossover pathway (class I crossovers) is thought to be MUS81-independent [62], [63]. However, our study suggests that MUS-81 may be able to make a portion of the MSH-4- and MSH-5-dependent crossovers in certain mutant situations in *C. elegans*.

In summary, we have addressed the functional overlaps and differences between these four structure-specific endonucleases regarding their regulatory roles in crossover control during meiosis. This has revealed novel roles for MUS-81, SLX-1 and XPF-1 in promoting the obligate crossover, and SLX-1 in regulating crossover distribution.

## Materials and Methods

### *C. elegans* genetics

*C. elegans* strains were cultured at 20°C under standard conditions [64]. The strains used in this study are listed in Table S4.

### Determining crossover frequencies and distribution

Meiotic crossover frequencies and distribution were assayed utilizing single-nucleotide polymorphism (SNP) markers as in [34]. The SNP markers located at the boundaries of the chromosome domains were chosen based on data from WormBase (WS231) and [26]. The SNP markers and primers used are listed in Table S5. PCR and restriction digests of single worm lysates were performed as described in [65]. Statistical analysis was performed using the two-tailed Fisher's Exact test and Chi square test, 95% C.I., as in [28], [66], and corrected by Sidak adjustment for multiple comparisons.

### Yeast two-hybrid analysis

The yeast two-hybrid assay was performed according to [67]. Full-length cDNAs for HIM-18, SLX-1, XPF-1, ERCC-1, MUS-81, EME-1, GEN-1, and RAD-54 were cloned into a Gateway donor vector (pDONR223). Each construct was then subcloned into 2μ Gateway destination vectors pVV213 (activation domain (AD), *LEU2*+) and pVV212 (Gal4 DNA binding domain (DB), *TRP1*+). AD-Y and DB-X fusions were transformed into *MAT*a Y8800 and *MAT*α Y8930 yeast strains, respectively. *MAT*a Y8800 and *MAT*α Y8930 were mated on YPD plates and diploids carrying both plasmids were selected on SC-Leu-Trp plates. The interactions were assessed by growth on SC-Leu-Trp-His plates at 30°C.

### Immunofluorescence and imaging

Whole-mounted dissected germ lines from adult hermaphrodites 21–24 h post-L4 larval stage were subjected to 1% formaldehyde fixation, as described in [18], with the exception of the addition of cold water fish skin gelatin (0.1%; Sigma) in the 1% BSA blocking solution. The following primary antibodies were used at the indicated dilutions: α-REC-8 (1∶100; Abcam), α-AIR-2 (1∶100; [68]), and α-ZHP-3 (1∶500; [23]).

Images were acquired at 100× magnification with or without 1.5× auxiliary magnification as stacks of optical sections at 0.2-µm intervals using an IX-70 microscope (Olympus) and a cooled CCD camera (model CH350; Roper Scientific) controlled by the DeltaVision system (Applied Precision). Images were subjected to deconvolution analysis using the SoftWorx Suite 3.0 program (Applied Precision) using an enhanced ratio algorithm with 15 iterations. Statistical analysis of ZHP-3 foci was performed by the two-tailed Mann-Whitney Test, 95% C.I., and corrected by Sidak adjustment for multiple comparisons.

## Supporting Information

Figure S1 Chromosome morphology defects observed in the oocytes of the structure-specific endonuclease-deficient mutants. Images from Figure 4B separated into individual channels. (A) A wild type −1 oocyte at diakinesis has no observable chromosomal defects by analysis of REC-8 (red) and AIR-2 (green) immunolocalization and DAPI-stained chromatin (blue). (B) Oocyte that exhibits an intrabivalent chromatin bridge. A chromatin bridge between homologs (red arrowhead) is observed in a −1 oocyte from an *slx-1; xpf-1* double mutant germline. Inset shows magnified image of the intrabivalent chromatin bridge. (C) An example of an interbivalent chromatin bridge (blue arrowhead) in a −1 oocyte from an *slx-1; xpf-1* double mutant germline. The chromatin bridge between bivalents is shown at a higher magnification in the inset. (D) An *slx-1; xpf-1* double mutant −1 oocyte shown with two pairs of homologs separated at their short arms (orange and purple arrowheads). The separated homologs (dissociated bivalent), as suggested by the uncoupling of the two AIR-2 rings (green), normally observed at the region of contact between the short arms, and the lack of observable DAPI-staining chromatin between separated short arms. Purple arrowhead points to the dissociated bivalent magnified in the inset. (E) One DNA fragment (green arrowhead) has separated from chromosomes in a −1 oocyte from an *slx-1* mutant germline. Inset depicts a higher magnification of the DNA fragment indicated by the green arrowhead. (F) A −1 oocyte from an *slx-1; gen-1* germline with frayed bivalents (yellow arrowhead). The inset shows a magnified single frayed bivalent indicated by the yellow arrowhead. Scale bars, 5 µm.
(TIF)

Table S1 Summary of the plate phenotypes.
(DOCX)

Table S2 Nominal P-values for the crossover analysis.
(DOCX)

Table S3 Corrected P-values for crossover analysis.
(DOCX)

Table S4 Strains used in this study.
(DOCX)

Table S5 SNP markers and primers used in the snip-SNP analysis.
(DOCX)
