# Supplementary figures and images for: Interplay between Structure-Specific Endonucleases for Crossover Control during Caenorhabditis elegans Meiosis
Source: PLoS Genet. 2013 Jul 18;9(7):e1003586. doi: 10.1371/journal.pgen.1003586 (PMC3715419; doi:10.1371/journal.pgen.1003586)

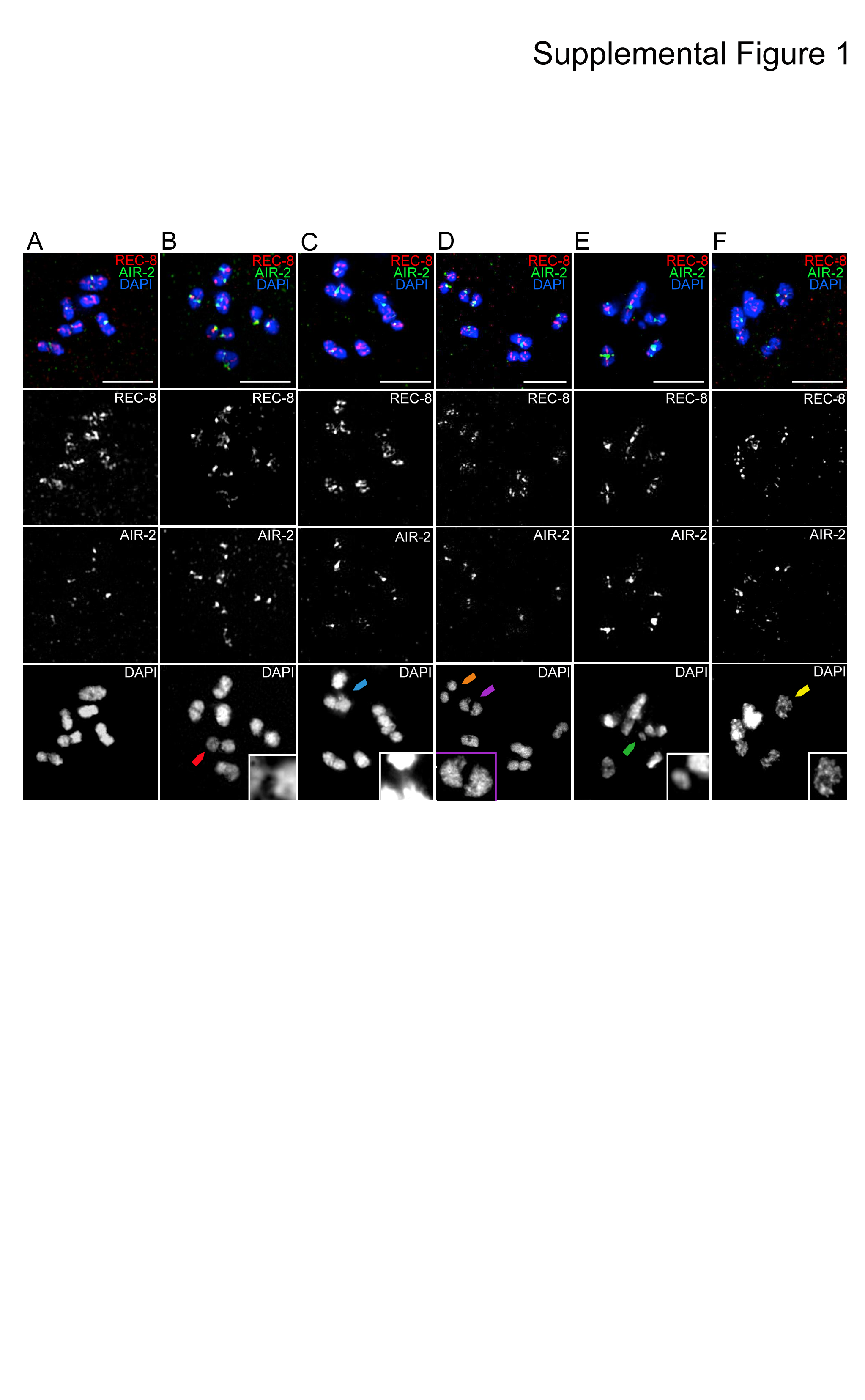

Supplement: Figure S1 — Chromosome morphology defects observed in the oocytes of the structure-specific endonuclease-deficient mutants. Images from Figure 4B separated into individual channels. (A) A wild type −1 oocyte at diakinesis has no observable chromosomal defects by analysis of REC-8 (red) and AIR-2 (green) immunolocalization and DAPI-stained chromatin (blue). (B) Oocyte that exhibits an intrabivalent chromatin bridge. A chromatin bridge between homologs (red arrowhead) is observed in a −1 oocyte from an slx-1; xpf-1 double mutant germline. Inset shows magnified image of the intrabivalent chromatin bridge. (C) An example of an interbivalent chromatin bridge (blue arrowhead) in a −1 oocyte from an slx-1; xpf-1 double mutant germline. The chromatin bridge between bivalents is shown at a higher magnification in the inset. (D) An slx-1; xpf-1 double mutant −1 oocyte shown with two pairs of homologs separated at their short arms (orange and purple arrowheads). The separated homologs (dissociated bivalent), as suggested by the uncoupling of the two AIR-2 rings (green), normally observed at the region of contact between the short arms, and the lack of observable DAPI-staining chromatin between separated short arms. Purple arrowhead points to the dissociated bivalent magnified in the inset. (E) One DNA fragment (green arrowhead) has separated from chromosomes in a −1 oocyte from an slx-1 mutant germline. Inset depicts a higher magnification of the DNA fragment indicated by the green arrowhead. (F) A −1 oocyte from an slx-1; gen-1 germline with frayed bivalents (yellow arrowhead). The inset shows a magnified single frayed bivalent indicated by the yellow arrowhead. Scale bars, 5 µm. (TIF) [file pgen.1003586.s001.tif]
