# Supplementary material for: Interplay between Structure-Specific Endonucleases for Crossover Control during Caenorhabditis elegans Meiosis
Source: PLoS Genet. 2013 Jul 18;9(7):e1003586. doi: 10.1371/journal.pgen.1003586 (PMC3715419; doi:10.1371/journal.pgen.1003586)
Supplement: Table S1 — Summary of the plate phenotypes. (DOCX) [file pgen.1003586.s002.docx]

**Table S1. Summary of the plate phenotypes**

| **Genotype** | **Mean no. of eggs/brood (n) ^a^** | **% Inviable embryos (n) ^b^** | **% Larval arrest (n) ^c^** | **% Males (n) ^d^** |
| --- | --- | --- | --- | --- |
| WT | 312 (11) | 1.6 (3435) | 1.7 (3379) | 0.2 (3321) |
| *mus-81* | 189 (15) | 7.0 (2769) | 3.6 (2575) | 0.4 (2483) |
| *slx-1 ^e^* | 211 (10) | 7.3 (2108) | 3.5 (1954) | 0.4 (1885) |
| *xpf-1* | 278 (16) | 7.7 (4444) | 1.3 (4102) | 1.5 (4049) |
| *gen-1* | 349 (10) | 0.6 (3486) | 0.7 (3465) | 0.03 (3440) |
| *mus-81 slx-1* | 184 (10) | 5.7 (1835) | 8.7 (1731) | 0.7 (1580) |
| *mus-81; xpf-1* | 180 (13) | 68.3 (2345) | 47.5 (743) | 8.5 (390) |
| *mus-81; gen-1* | 205 (15) | 3.6 (3071) | 3.1 (2960) | 0.5 (2869) |
| *slx-1; xpf-1* | 161 (13) | 50.8 (2095) | 28.3 (1031) | 3.1 (739) |
| *slx-1; gen-1* | 98 (11) | 9.4 (1083) | 18.5 (981) | 0.3 (800) |
| *xpf-1; gen-1* | 230 (15) | 3.2 (3451) | 2.0 (3340) | 2.0 (3273) |
| *mus-81 slx-1; xpf-1* | 149 (11) | 75.0 (1640) | 57.3 (410) | 6.3 (175) |
| *mus-81 slx-1; gen-1* | 219 (10) | 10.2 (2188) | 8.5 (1965) | 0.8 (1798) |
| *mus-81; xpf-1; gen-1(B)* | ND (20) | ND | ND | ND |
| *mus-81; xpf-1; gen-1(H)* | 152 (10) | 75.4 (1515) | 59.9 (372) | 8.1 (149) |
| *slx-1; xpf-1; gen-1* | 169 (5) | 34.2 (845) | 33.6 (556) | 6.0 (369) |
| *mus-81 slx-1; xpf-1; gen-1* | 142 (17) | 85.8 (2408) | 78.7 (342) | 4.1 (73) |
| *him-6 ^e^* | 252 (9) | 59.1 (2270) | 5.4 (928) | 13.7 (878) |
| *mus-81; him-6* | 54 (12) | 95.7 (650) | 92.9 (28) | ND (2) |
| *slx-1; him-6* | 49 (21) | 98.4 (981) | 93.8 (16) | ND (1) |
| *xpf-1;him-6* | 283 (10) | 57.4 (2829) | 9.7 (1205) | 14.3 (1088) |
| *gen-1;him-6* | 305 (10) | 55.1 (3048) | 9.5 (1369) | 14.5 (1239) |

Parentheses indicate the total number of: ^a^singled hermaphrodites for which entire brood sizes were scored, ^b^fertilized eggs scored, ^c^L1-L4 worms, ^d^adults scored, ^e^data from [18,21]. ND, not determined due to low n-value. B, Bristol and H, Hawaiian.
