# Supplementary material for: Interplay between Structure-Specific Endonucleases for Crossover Control during Caenorhabditis elegans Meiosis
Source: PLoS Genet. 2013 Jul 18;9(7):e1003586. doi: 10.1371/journal.pgen.1003586 (PMC3715419; doi:10.1371/journal.pgen.1003586)
Supplement: Table S2 — Nominal P-values for the crossover analysis. (DOCX) [file pgen.1003586.s003.docx]

**Table S2. Nominal P-values for the crossover analysis**

| **Chromosome V** | **Frequency** | | | | **Distribution** | | |
| --- | --- | --- | --- | --- | --- | --- | --- |
|  | a-d | a-b | b-c | c-d | a-b | b-c | c-d |
| *mus-81* | 1.0000 | 0.4498 | 0.2808 | 0.0876 | 0.3864 | 0.2453 | 0.0639 |
| *slx-1* | 0.8301 | 0.5620 | 0.0293 | 0.5048 | 0.4193 | 0.0312 | 0.2820 |
| *xpf-1* | 0.8192 | 0.1022 | 0.8485 | 0.1948 | 0.0933 | 1.0000 | 0.0956 |
| *gen-1* | 0.6710 | 0.2449 | 0.5867 | 0.0447 | 0.1467 | 0.4491 | 0.0351 |
| *mus-81 slx-1* | 0.9043 | 0.6274 | 0.0239 | 0.4533 | 0.4722 | 0.0260 | 0.2273 |
| *mus-81;xpf-1* | 0.0041 | 0.1428 | 0.6892 | 0.0731 | 1.0000 | 0.6142 | 0.8364 |
| *mus-81;gen-1* | 0.7316 | 0.5346 | 0.3532 | 0.6727 | 0.5948 | 0.2278 | 0.7369 |
| *slx-1;xpf-1* | 0.0133 | 0.2001 | 1.0000 | 0.0769 | 0.9017 | 0.3983 | 0.6337 |
| *slx-1;gen-1* | 1.0000 | 0.1498 | 1.0000 | 0.3114 | 0.1025 | 0.8566 | 0.1866 |
| *xpf-1;gen-1* | 0.7854 | 0.0534 | 0.0963 | 1.0000 | 0.0741 | 0.0800 | 0.8469 |
| *mus-81 slx-1;xpf-1* | 0.0870 | 0.5925 | 0.2834 | 0.0184 | 1.0000 | 0.0421 | 0.0817 |
| *mus-81 slx-1;gen-1* | 1.0000 | 0.5349 | 0.1924 | 0.7761 | 0.4840 | 0.1636 | 0.7368 |
| *mus-81;xpf-1;gen-1* | 0.0142 | 0.5776 | 1.0000 | 0.0373 | 0.8061 | 0.3895 | 0.2454 |
| *slx-1;xpf-1;gen-1* | 0.0019 | 0.1497 | 0.8957 | 0.0097 | 1.0000 | 0.1436 | 0.2560 |
| *mus-81 slx-1;xpf-1;gen-1* | 0.0002 | 0.3251 | 0.2048 | 0.0106 | 0.4395 | 1.0000 | 0.4547 |
|  |  |  |  |  |  |  |  |
| **X chromosome** | Frequency | | | | Distribution | | |
|  | a-d | a-b | b-c | c-d | a-b | b-c | c-d |
| *mus-81* | 0.1875 | 0.1651 | 0.5712 | 0.4686 | 0.4655 | 0.3338 | 1.0000 |
| *slx-1* | 0.2671 | 0.7948 | 0.4400 | 0.2793 | 0.2468 | 0.6713 | 0.4758 |
| *xpf-1* | 1.0000 | 0.7008 | 0.5853 | 0.6521 | 0.7581 | 0.6993 | 0.5079 |
| *gen-1* | 0.2412 | 0.2209 | 0.5934 | 0.8855 | 0.5640 | 0.8563 | 0.4411 |
| *mus-81 slx-1* | 0.5406 | 0.4688 | 0.3955 | 0.5159 | 0.7181 | 0.4913 | 0.2591 |
| *mus-81;xpf-1* | 4.85E-08 | 0.0799 | 0.0002 | 0.0019 | 0.0195 | 0.0281 | 0.4659 |
| *mus-81;gen-1* | 0.8176 | 0.3505 | 0.6875 | 1.0000 | 0.4208 | 0.5325 | 0.8636 |
| *slx-1;xpf-1* | 3.04E-05 | 9.43E-06 | 0.7984 | 0.1958 | 0.0102 | 0.0328 | 0.4510 |
| *slx-1;gen-1* | 0.3628 | 1.0000 | 0.5553 | 0.5326 | 0.6105 | 0.8277 | 0.8564 |
| *xpf-1;gen-1* | 0.8619 | 0.4144 | 0.7592 | 0.4839 | 0.2111 | 0.7434 | 0.5889 |
| *mus-81 slx-1;xpf-1* | 7.20E-06 | 0.0038 | 0.1720 | 0.1026 | 0.6020 | 1.0000 | 0.5810 |
| *mus-81 slx-1;gen-1* | 0.2876 | 0.6791 | 0.3279 | 1.0000 | 0.8735 | 0.4322 | 0.7347 |
| *mus-81;xpf-1;gen-1* | 0.0012 | 0.0924 | 0.0082 | 0.7030 | 1.0000 | 0.0797 | 0.1819 |
| *slx-1;xpf-1;gen-1* | 0.0010 | 1.60E-06 | 0.3425 | 0.7926 | 0.0002 | 0.0141 | 2.07E-05 |
| *mus-81 slx-1;xpf-1;gen-1* | 4.04E-07 | 0.00002 | 0.0082 | 0.7030 | 0.0399 | 0.3684 | 0.0013 |

Highlighted cells indicate statistical difference compared to wild type (blue; P<0.05, grey; P<0.01, and yellow; P<0.001 by the Fisher’s Exact Test.
