# Supplementary material for: Interplay between Structure-Specific Endonucleases for Crossover Control during Caenorhabditis elegans Meiosis
Source: PLoS Genet. 2013 Jul 18;9(7):e1003586. doi: 10.1371/journal.pgen.1003586 (PMC3715419; doi:10.1371/journal.pgen.1003586)
Supplement: Table S3 — Corrected P-values for crossover analysis. (DOCX) [file pgen.1003586.s004.docx]

**Table S3. Corrected P-values for crossover analysis**

| **Chromosome V** | **Frequency** | | | | **Distribution** | | |
| --- | --- | --- | --- | --- | --- | --- | --- |
|  | a-d | a-b | b-c | c-d | a-b | b-c | c-d |
| *mus-81* | 1.0000 | 0.9999 | 0.9929 | 0.7472 | 0.9993 | 0.9853 | 0.6286 |
| *slx-1* | 1.0000 | 1.0000 | **0.3599** | 1.0000 | 0.9997 | **0.3784** | 0.9931 |
| *xpf-1* | 1.0000 | 0.8015 | 1.0000 | 0.9612 | 0.7699 | 1.0000 | 0.7785 |
| *gen-1* | 1.0000 | 0.9852 | 1.0000 | **0.4964** | 0.9074 | 0.9999 | **0.4149** |
| *mus-81 slx-1* | 1.0000 | 1.0000 | **0.3043** | 0.9999 | 0.9999 | **0.3264** | 0.9791 |
| *mus-81;xpf-1* | **0.0598** | 0.9009 | 1.0000 | 0.6797 | 1.0000 | 1.0000 | 1.0000 |
| *mus-81;gen-1* | 1.0000 | 1.0000 | 0.9985 | 1.0000 | 1.0000 | 0.9793 | 1.0000 |
| *slx-1;xpf-1* | **0.1820** | 0.9649 | 1.0000 | 0.6989 | 1.0000 | 0.9995 | 1.0000 |
| *slx-1;gen-1* | 1.0000 | 0.9123 | 1.0000 | 0.9963 | 0.8025 | 1.0000 | 0.9549 |
| *xpf-1;gen-1* | 1.0000 | 0.5610 | 0.7810 | 1.0000 | 0.6849 | 0.7137 | 1.0000 |
| *mus-81 slx-1;xpf-1* | 0.7447 | 1.0000 | 0.9933 | **0.2431** | 1.0000 | **0.4754** | 0.7215 |
| *mus-81 slx-1;gen-1* | 1.0000 | 1.0000 | 0.9595 | 1.0000 | 1.0000 | 0.9314 | 1.0000 |
| *mus-81;xpf-1;gen-1* | **0.1931** | 1.0000 | 1.0000 | **0.4346** | 1.0000 | 0.9994 | 0.9854 |
| *slx-1;xpf-1;gen-1* | **0.0281** | 0.9122 | 1.0000 | **0.1360** | 1.0000 | 0.9022 | 0.9882 |
| *mus-81 slx-1;xpf-1;gen-1* | **0.0030** | 0.9973 | 0.9679 | **0.1477** | 0.9998 | 1.0000 | 0.9999 |
|  |  |  |  |  |  |  |  |
| **X chromosome** | **Frequency** | | | | **Distribution** | | |
|  | a-d | a-b | b-c | c-d | a-b | b-c | c-d |
| *mus-81* | 0.9556 | 0.9332 | 1.0000 | 0.9999 | 0.9999 | 0.9977 | 1.0000 |
| *slx-1* | 0.9905 | 1.0000 | 0.9998 | 0.9926 | 0.9858 | 1.0000 | 0.9999 |
| *xpf-1* | 1.0000 | 1.0000 | 1.0000 | 1.0000 | 1.0000 | 1.0000 | 1.0000 |
| *gen-1* | 0.9841 | 0.9763 | 1.0000 | 1.0000 | 1.0000 | 1.0000 | 0.9998 |
| *mus-81 slx-1* | 1.0000 | 0.9999 | 0.9995 | 1.0000 | 1.0000 | 1.0000 | 0.9889 |
| *mus-81;xpf-1* | **<0.0001** | 0.7132 | **0.0030** | **0.0281** | **0.2558** | **0.3479** | 0.9999 |
| *mus-81;gen-1* | 1.0000 | 0.9985 | 1.0000 | 1.0000 | 0.9997 | 1.0000 | 1.0000 |
| *slx-1;xpf-1* | **0.0005** | **0.0001** | 1.0000 | 0.9619 | **0.1425** | **0.3936** | 0.9999 |
| *slx-1;gen-1* | 0.9988 | 1.0000 | 1.0000 | 1.0000 | 1.0000 | 1.0000 | 1.0000 |
| *xpf-1;gen-1* | 1.0000 | 0.9997 | 1.0000 | 1.0000 | 0.9715 | 1.0000 | 1.0000 |
| *mus-81 slx-1;xpf-1* | **0.0001** | **0.0555** | 0.9411 | 0.8029 | 1.0000 | 1.0000 | 1.0000 |
| *mus-81 slx-1;gen-1* | 0.9938 | 1.0000 | 0.9974 | 1.0000 | 1.0000 | 0.9998 | 1.0000 |
| *mus-81;xpf-1;gen-1* | **0.0178** | 0.7664 | **0.1162** | 1.0000 | 1.0000 | 0.7123 | 0.9508 |
| *slx-1;xpf-1;gen-1* | **0.0149** | **<0.0001** | 0.9981 | 1.0000 | **0.0031** | **0.1915** | **0.0003** |
| *mus-81 slx-1;xpf-1;gen-1* | **<0.0001** | **0.0002** | **0.1162** | 1.0000 | **0.4571** | 0.9990 | **0.0193** |

Highlighted cells indicate statistical difference compared to wild type (blue; P<0.05, grey; P<0.01, and yellow; P<0.001 by the Fisher’s Exact Test and corrected by Sidak adjustment for multiple comparisons). Bold type indicates statistical significance (P<0.05) before Sidak correction. Sidak correction was calculated by the following formula: Pc = 1-(1-P)^k^; Pc = corrected P-value, P = nominal P-value, k = number of comparisons. In the table, k=15.
