# Supplementary material for: Interplay between Structure-Specific Endonucleases for Crossover Control during Caenorhabditis elegans Meiosis
Source: PLoS Genet. 2013 Jul 18;9(7):e1003586. doi: 10.1371/journal.pgen.1003586 (PMC3715419; doi:10.1371/journal.pgen.1003586)
Supplement: Table S4 — Strains used in this study. (DOCX) [file pgen.1003586.s005.docx]

**Table S4. Strains used in this study**

| **Strain** | **Genotype** |
| --- | --- |
| N2 | wild type (Bristol) |
| CV170 | *mus-81(tm1937) I / hT2[bli-4(e937) let-?(q782) qIs48] (I;III)* |
| CV171 | *slx-1(tm2644) I / hT2[bli-4(e937) let-?(q782) qIs48] (I;III)* |
| CV168 | *xpf-1(e1487) / mIn1[dpy-10(e128) mIs14] II* |
| CV186 | *gen-1(tm2940) III* |
| CV294 | *mus-81(tm1937)slx-1(tm2644) I / hT2[bli-4(e937) let-?(q782) qIs48] (I;III)* |
| CV214 | *mus-81(tm1937) I; xpf-1(e1487) / mIn1[dpy-10(e128) mIs14] II* |
| CV289 | *mus-81(tm1937) I / hT2[bli-4(e937) let-?(q782) qIs48] (I;III); gen-1(tm2940) III / hT2[bli-4(e937) let-?(q782) qIs48] (I;III)* |
| CV215 | *slx-1(tm2644) I ; xpf-1(e1487) / mIn1[dpy-10(e128) mIs14] II* |
| CV212 | *slx-1(tm2644) I / hT2[bli-4(e937) let-?(q782) qIs48] (I;III); gen-1(tm2940) III / hT2[bli-4(e937) let-?(q782) qIs48] (I;III)* |
| CV290 | *xpf-1(e1487) / mIn1[dpy-10(e128) mIs14] II; gen-1(tm2940) III* |
| CV296 | *mus-81(tm1937)slx-1(tm2644) I ; xpf-1(e1487) / mIn1[dpy-10(e128) mIs14] II* |
| CV297 | *mus-81(tm1937)slx-1(tm2644) I / hT2[bli-4(e937) let-?(q782) qIs48] (I;III); gen-1(tm2940) III / hT2[bli-4(e937) let-?(q782) qIs48] (I;III)* |
| CV292 | *mus-81(tm1937) I; xpf-1(e1487) / mIn1[dpy-10(e128) mIs14] II; gen-1(tm2940) III* |
| CV285 | *slx-1(tm2644) I; xpf-1(e1487) / mIn1[dpy-10(e128) mIs14] II; gen-1(tm2940) III* |
| CV298 | *mus-81(tm1937)slx-1(tm2644) I; xpf-1(e1487) / mIn1[dpy-10(e128) mIs14] II; gen-1(tm2940) III* |
| CV120 | *ccIs4251* |
| CV123 | *him-6(ok412)/nT1[qIs51] (VI;V)* |
| CV304 | *mus-81(tm1937) I; him-6(ok412)/nT1[qIs51] (VI;V)* |
| CV311 | *slx-1(tm2644) I; him-6(ok412)/nT1[qIs51] (VI;V)* |
| CV211 | *xpf-1(e1487) / mIn1[dpy-10(e128) mIs14] II; him-6(ok412)/nT1[unc-?(n754) let-?] (VI;V)* |
| CV302 | *gen-1(tm2940) III; him-6(ok412)/nT1[qIs51] (VI;V)* |
| CB4856 | wild type (Hawaiian) |
| CV222 | *mus-81(tm1937) I / hT2[bli-4(e937) let-?(q782) qIs48] (I;III)* (Hawaiian) |
| CV212 | *slx-1(tm2644) I / hT2[bli-4(e937) let-?(q782) qIs48] (I;III)* (Hawaiian) |
| CV229 | *xpf-1(e1487) / mIn1[dpy-10(e128) mIs14] II* (Hawaiian) |
| CV299 | *gen-1(tm2940) III* (Hawaiian) |
| CV300 | *mus-81(tm1937)slx-1(tm2644) I / hT2[bli-4(e937) let-?(q782) qIs48] (I;III)* (Hawaiian) |
| CV283 | *mus-81(tm1937) I; xpf-1(e1487) / mIn1[dpy-10(e128) mIs14] II* (Hawaiian) |
| CV306 | *mus-81(tm1937) I / hT2[bli-4(e937) let-?(q782) qIs48] (I;III); gen-1(tm2940) III / hT2[bli-4(e937) let-?(q782) qIs48] (I;III)* (Hawaiian) |
| CV282 | *slx-1(tm2644) I ; xpf-1(e1487) / mIn1[dpy-10(e128) mIs14] II* (Hawaiian) |
| CV307 | *slx-1(tm2644) I / hT2[bli-4(e937) let-?(q782) qIs48] (I;III); gen-1(tm2940) III / hT2[bli-4(e937) let-?(q782) qIs48] (I;III)* (Hawaiian) |
| CV301 | *xpf-1(e1487) / mIn1[dpy-10(e128) mIs14] II; gen-1(tm2940) III* (Hawaiian) |
| CV316 | *mus-81(tm1937)slx-1(tm2644) I ; xpf-1(e1487) / mIn1[dpy-10(e128) mIs14] II* (Hawaiian) |
| CV312 | *mus-81(tm1937)slx-1(tm2644) I / hT2[bli-4(e937) let-?(q782) qIs48] (I;III); gen-1(tm2940) III / hT2[bli-4(e937) let-?(q782) qIs48] (I;III)* (Hawaiian) |
| CV329 | *mus-81(tm1937) I; xpf-1(e1487) / mIn1[dpy-10(e128) mIs14] II; gen-1(tm2940) III* (Hawaiian) |
| CV303 | *slx-1(tm2644) I; xpf-1(e1487) / mIn1[dpy-10(e128) mIs14] II; gen-1(tm2940) III* (Hawaiian) |
| CV305 | *mus-81(tm1937)slx-1(tm2644) I; xpf-1(e1487) / mIn1[dpy-10(e128) mIs14] II; gen-1(tm2940) III* (Hawaiian) |

The integrated transgenes are *qIs48[Pmyo-2::gfp; Ppes-10::gfp; Pges-1::gfp], qIs26[lag-2::GFP; rol-6(d)]III, qIs51[myo-2::GFP; pes-10::GFP; F22B7.9::GFP], mIs14[myo-2::gfp; pes-10::gfp]II,* and *ccIs4251[myo-3::Ngfp-lacZ; myo-3::Mtgfp]I.*
