# Supplementary material for: Interplay between Structure-Specific Endonucleases for Crossover Control during Caenorhabditis elegans Meiosis
Source: PLoS Genet. 2013 Jul 18;9(7):e1003586. doi: 10.1371/journal.pgen.1003586 (PMC3715419; doi:10.1371/journal.pgen.1003586)
Supplement: Table S5 — SNP markers and primers used in the snip-SNP analysis. (DOCX) [file pgen.1003586.s006.docx]

**Table S5. SNP markers and primers used in the snip-SNP analysis**

| Chr. | Position (kb) | SNP | Primers | Enzyme | Bristol digest (bp) | Hawaiian digest (bp) |
| --- | --- | --- | --- | --- | --- | --- |
| V | A  (537) | pKP5098 | TGTAGGGCGAGTAACCAAGC  CCGCACTTCCTTCAGAAATG | *Bam*HI | 318 | 268, 50 |
|  | B  (6,043) | pKP5059 | TTCGGTGTGTTGTCTGTAGTCG  GTTGTGAAAGTCGCGTTGAG | *Bsp*HI | 320, 207 | 527 |
|  | C (16,577) | pKP5130 | AGCCGGGTAGAGAGCTAAAGTG  TTCATGACAAGCATCCAGTGTC | *Hpy*CH4IV | 215, 33 | 197, 33, 18 |
|  | D (20,760) | pKP5075 | TCCCCTCTACCAGATGCCTT  ACTGCTAGCTCAAATACTCCCA | *Dra*I | 374, 101 | 475 |
| X | A  (535) | pKP6100 | TGGCAAAACACATCCCTGTG  GGTATCCGATCCCTTCAACAAG | *Bsp*HI | 208, 156 | 364 |
|  | B  (6,152) | pKP6108 | AGCAATCTGGATATGCAAATCC  GAATACTCGGAGCGGTGCCA | *Nsi*I | 488, 85 | 573 |
|  | C (12,208) | pKP6125 | ACAGTAAGATGACCATACACACG  AAGCAGCGCGAGGTATGTAG | *Taq*I | 308 | 210, 98 |
|  | D (17,701) | pKP6172 | TTCTGTTGATTTGGTTGCTCCG  TGATGCAGGAACAAAAGTAGTG | *Apo*I | 174, 117 | 291 |
